# ﻿The ectoparasitoid wasp *Heterospilussicanus* (Marshall, 1888) (Hymenoptera, Braconidae, Doryctinae) as a natural enemy of *Gastralluspubens* Fairmaire, 1875 (Coleoptera, Ptinidae) in Italy

**DOI:** 10.3897/zookeys.1201.118549

**Published:** 2024-05-14

**Authors:** Sergey A. Belokobylskij, Salvatore Guarino, Sara Savoldelli, Costanza Jucker, Ezio Peri, Gavin R. Broad, Giuliano Cerasa

**Affiliations:** 1 Zoological Institute of the Russian Academy of Sciences, St Petersburg 199034, Russia Zoological Institute of the Russian Academy of Sciences St Petersburg Russia; 2 Institute of Biosciences and Bioresources (IBBR), National Research Council of Italy (CNR), Corso Calatafimi 414, 90129 Palermo, Italy Institute of Biosciences and Bioresources (IBBR), National Research Council of Italy (CNR) Palermo Italy; 3 DeFENS – Department of Food, Environmental and Nutritional Sciences, University of Milan, Via G. Celoria 2, 20133 Milan, Italy University of Milan Milan Italy; 4 Department of Agriculture, Food and Forest Sciences, University of Palermo, Viale delle Scienze, Building 5, 90128 Palermo, Italy University of Palermo Palermo Italy; 5 Natural History Museum, Cromwell Road, London SW7 5BD, UK The Natural History Museum London United Kingdom

**Keywords:** Bookworm, diagnosis, Ichneumonoidea, parasitoid, redescriptions, taxonomy

## Abstract

*Heterospilussicanus* (Marshall, 1888) is redescribed and illustrated based on the holotype of *Dendrosotersicanus* Marshall, 1888 and on recently collected material from its type locality (Sicily, Italy). Previous host records for this species are unreliable. Here, the host of *H.sicanus*, the rare ptinid beetle *Gastralluspubens* Fairmaire, 1875, is recorded for the first time, having been reared in a historic library in Palermo, Italy. *Heterospilussicanus* is compared with the similar species *Telebolus* (= *Heterospilus*) *corsicus* Marshall, 1888, which was described in the same monograph from Corsica (France), and it is also redescribed and illustrated. *Atoreuteusceballosi* Docavo Alberti, 1960, **syn. nov.** is synonymised under *Heterospilussicanus* (Marshall, 1888), and *Hormiopterus* (= *Rhaconotus*) *ollivieri* Giraud var. flava Fahringer, 1931, **syn. nov.** is a junior synonym of *Heterospiluscephi* Rohwer, 1925. A key for determination of the Western Palaearctic *Heterospilus* species with a striate vertex is provided and the distributions of *H.sicanus* and *H.corsicus* are discussed.

## ﻿Introduction

The family Braconidae is a vast group within the order Hymenoptera, comprising more than 20,000 recognised species (Yu et al. 2016). Together with the family Ichneumonidae it constitutes one of two recent families of Ichneumonoidea, the vast majority of which are parasitoids (Wharton 1993).

The braconid subfamily Doryctinae is renowned for its exceptional genera and species richness and diversity, including more than 2000 described species distributed globally across almost 200 genera (Shenefelt and Marsh 1976; Marsh 1993, 2002; Belokobylskij et al. 2004a, 2004b; Belokobylskij and Maetô 2009; Yu et al. 2016). Doryctines, for the most part, are idiobiont ectoparasitoids on the larvae of xylophagous and bark-boring Coleoptera. Some members of this group also parasitise the larvae of Lepidoptera and Hymenoptera (Symphyta), while a few genera are known to be phytophagous (Ramírez and Marsh 1996; Wharton and Hanson 2005; Zaldívar-Riverón et al. 2007, 2014). Additionally, in certain cases, they function as parasitoids (perhaps endoparasitoids) of adult Embioptera or have been observed inhabiting termite nests (Shaw and Edgerly 1985; Wharton 1993; Kistner et al. 2000; Belokobylskij 2002).

Within this subfamily, the genus *Heterospilus* Haliday, 1836, belonging to the tribe Heterospilini, stands out as one of the largest and most hyperdiverse braconid genera, with already more than 400 species described and many more to be described (Marsh et al. 2013; Yu et al. 2016; Ghahari et al. 2022). In total, 21 species of *Heterospilus* of 45 Palaearctic species are known in Europe, while more than 340 species have been described from the New World (Nearctic and mainly Neotropics) and 37 species from the Oriental region; only one species has been described from Australia and none from the Afrotropical region (Yu et al. 2016; Belokobylskij and Ku 2021).

Species of *Heterospilus* are idiobiont ectoparasitoids known for their exceptionally diverse range of primarily endophytic hosts (Belokobylskij and Maeto 2009; Yu et al. 2016), primarily targeting stem-boring Coleoptera of various families, including Anobiidae, Buprestidae, Cerambycidae, Chrysomelidae (mainly Bruchinae), Curculionidae (including Scolytinae), Languriidae, Mordellidae, and Ptinidae. Additionally, they also parasitise Lepidoptera species of the families Cosmopterigidae, Gelechiidae, Prodoxidae and Pyralidae, and even stem-boring Hymenoptera of the family Cephidae. In addition, a few species have been reared from nests of Crabronidae (Hymenoptera) (Marsh 1982; Shaw 1995; Marsh and Melo 1999; Cabrera et al. 2002).

In this study we provide an illustrated redescription and updated diagnosis of *Heterospilussicanus* (Marshall, 1888), discovered in the frass, holes, and tunnels created by *Gastralluspubens* Fairmaire, 1875 (Coleoptera: Ptinidae) in books seriously infested by this book-boring beetle during inspections in the “Ottavio Ziino” Law History Library of the Law Department at the University of Palermo (Sicily, Italy). *Heterospilussicanus* is compared with the congeneric *Telebolus* (= *Heterospilus*) *corsicus* Marshall, 1888, which is also redescribed and illustrated, and *Atoreuteusceballosi* Docavo Alberti, 1960 is here synonymised under *H.sicanus* (Marshall, 1888). Finally, a key for the determination of the Western Palaearctic *Heterospilus* species with a striate vertex is included and the distributions of *H.sicanus* and *H.corsicus* are discussed.

## ﻿Materials and methods

The terminology employed in this work for the morphological features, measurements, and wing venation nomenclature follows Belokobylskij and Maeto (2009), with the terminology for wing venation by van Achterberg (1993) shown in parentheses. Images were taken using a Leica DM series compound microscope (Leica, Benzheim, Germany) and a Leica DFC series mounted camera with Leica Application Suite software (LAS EZ 3.4.0, Leica, Switzerland), and with a Canon SLR EOS 5DSR with either a 65 mm macro lens or a Mitutoyo 10× lens in combination with a 70–130 mm macro lens, mounted on a stand with an automated Z-stepper (the Natural History Museum, London, UK). All insect photos were integrated using the freeware CombineZP (Hadley 2011) or Helicon Focus and processed in Adobe Photoshop CS4.

### ﻿Abbreviations of specimen depositories

The specimens (including types) examined in this study have been deposited in the following collections.

**HNHM**Hungarian Natural History Museum, Budapest, Hungary;

**MNCN**Museo Nacional de Ciencias Naturales, Madrid, Spain;

**NHMUK**the Natural History Museum, London, UK;

**NHMW**Naturhistorisches Museum, Wien, Austria;

**SAAF-UNIPA** Department of Agricultural, Food and Forest Science, University of Palermo, Palermo, Italy;

**ZISP**Zoological Institute, Russian Academy of Sciences, St Petersburg, Russia.

## ﻿Taxonomy

### ﻿Class Hexapoda Blainville, 1816

**Order Hymenoptera Linnaeus, 1758**

**Family Braconidae Nees, 1811**

**Subfamily Doryctinae Foerster, 1863**

**Tribe Heterospilini Fischer, 1981**

#### Genus *Heterospilus* Haliday, 1836

The original description of *Dendrosotersicanus* Marshall, 1888: 242, translated from French, is as follows:

“Head transverse, largely dark brown as well as the thorax; the rest of the body tawny; abdomen black towards the tip. Vertex high, gibbous, without frontal protuberances, finely wrinkled crosswise; eye and stemmaticum smooth. Ocelli sunk in the head, the front one placed on the slope of the forehead. Frontal excavation very shallow and poorly determined. Orbits and genae fawn. Antennae as long as the body, slender, blackish with ferruginous base, with 20 antennal segments. Thorax granular, slightly shiny. Mesonotum dark brown; its crenulated furrows converging towards a deep, rough dimple. Metanotum fawn, slightly shiny, granular irregularly streaked lengthwise on its anterior part, roughly reticulated in rear, with several high lines which cross in all directions. Wings slightly smoky, veins and stigma brown; second cubital cell receiving the recurrent vein; vein posterior non-interstitial. Legs fairly short and thick, testaceous. Abdomen as long as the head and thorax, and wider than the latter, tawny, becoming more and more blackish towards the end, last segment pale; first segment in truncated triangle, twice wider at the tip than at the base, bicarinated and high in the middle, depressed on the side edges, leathery, dull, longitudinally streaked. Second suture erased, even on the sides. Second segment very linearly wrinkled at the base, smooth and shiny on the rest of its surface, as well as all the following ones. Ovipositor as long as the abdomen. Male unknown. Long. 2–3.5 mm.”

Marshall (1897: 127) additionally noted (here translated from French), “This species [*D.sicanus*], like the others, is variable as to the size and the colours. I received from Genoa two ♀ which are much darker than the type, and one of which is only half the size indicated. In other aspects their features agree with those of *D.sicanus*. Homeland: add, Italy (Genoa), sent from Mr Mantero.”

Mantero (1904: 28) elaborated on the Italian specimens (here translated from Italian): “Belvedere, July 1891 (Solari). The Ligurian specimens, also cited by Marshall (1897) have a darker colour than the type.”

##### ﻿ Heterospilus sicanus

Taxon classificationAnimalia

(Marshall, 1888)

92C003FE-9189-5218-8085-9B1A1E66226E

**Figure 1.**
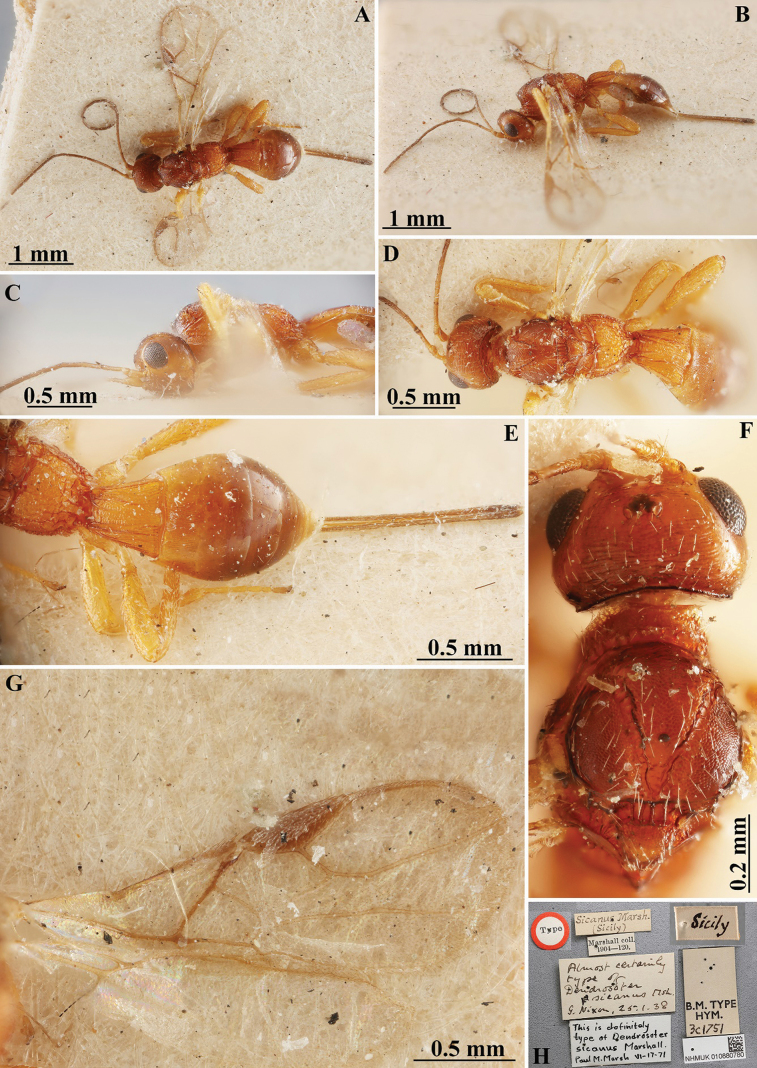
*Heterospilussicanus* (Marshall, 1888) (female, holotype) **A** habitus, dorsal view **B** habitus, lateral view **C** head, mesosoma and base of metasoma, lateral view **D** head, mesosoma and base of metasoma, dorsal view **E** propodeum, metasoma and ovipositor, dorsal view **F** head and mesoscutum, dorsal view **G** wings **H** labels.

**Figure 2.**
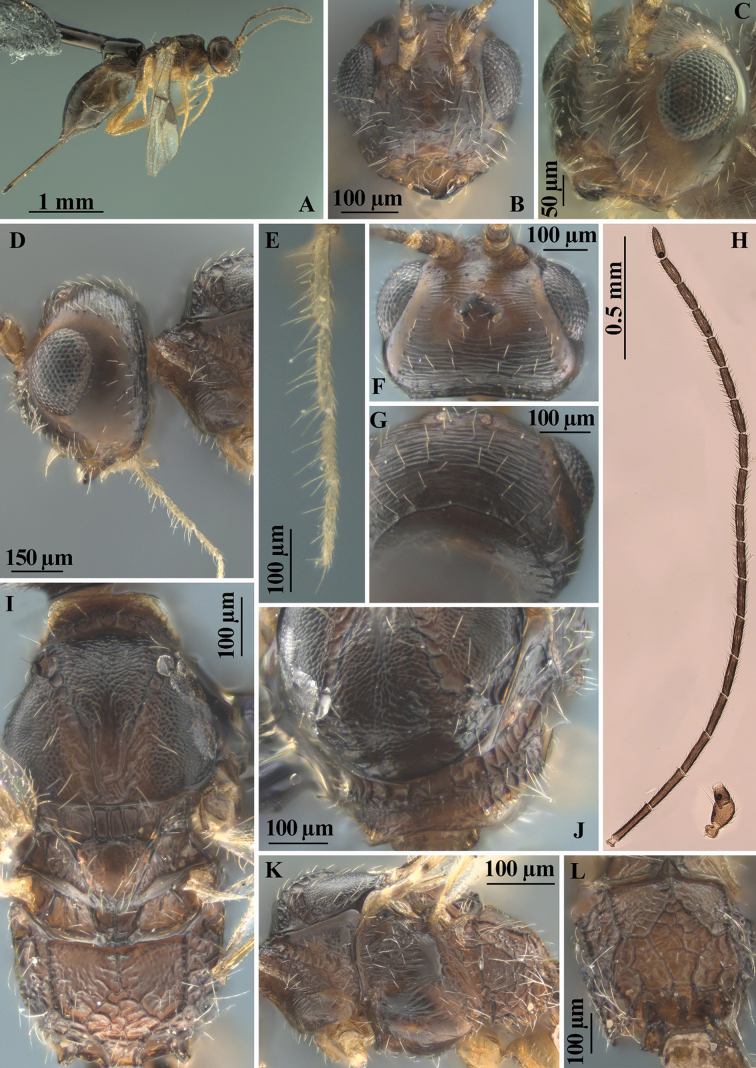
*Heterospilussicanus* (Marshall, 1888) (female) **A** habitus, lateral view **B–D** head, frontal, antero-lateral and lateral view **E** maxillary palp **F, G** head, dorsal and postero-dorsal view **H** antenna **I** mesosoma, dorsal view **J** pronotum, antero-dorsal view **K** mesosoma, lateral view **L** propodeum, dorsal view.

**Figure 3.**
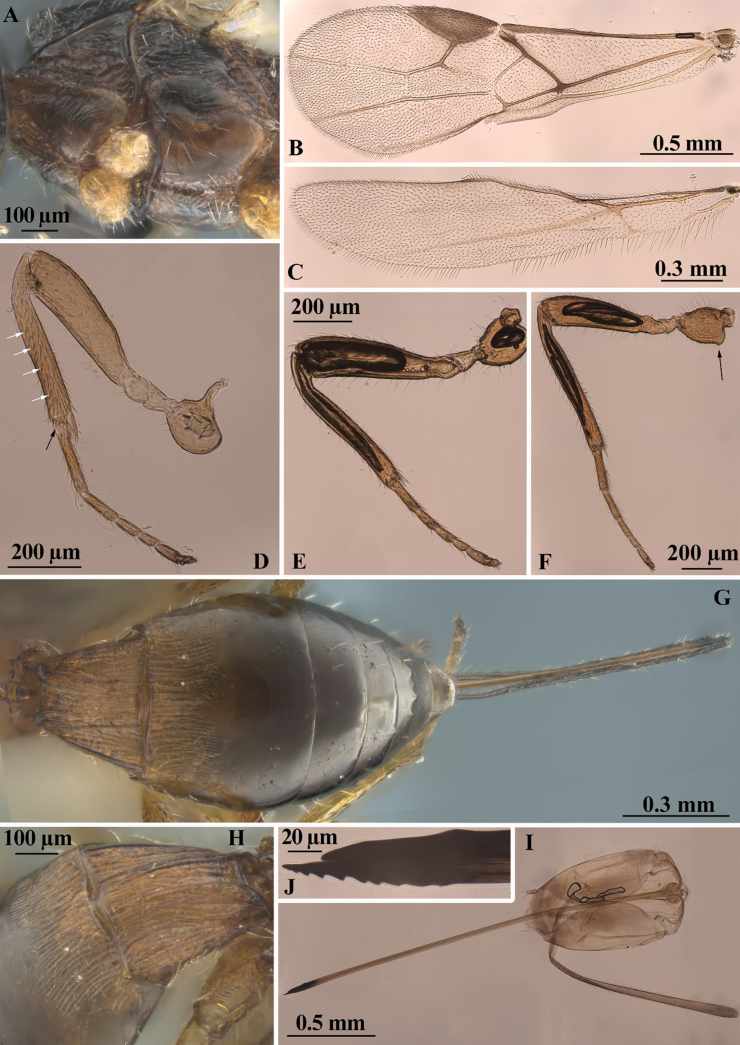
*Heterospilussicanus* (Marshall, 1888) (female) **A** mesosoma, ventro-lateral view **B** fore wing **C** hind wing **D** fore leg (white arrows indicate short stout spines on its front tibia surface and black arrow those on the apical part) **E** middle leg **F** hind leg (black arrow indicates a distinct antero-ventral basal tubercle on the coxa) **G** metasoma, dorsal view **H** first tergite, dorso-lateral view **I** ovipositor and one of its sheaths **J** ovipositor apex.

**Figure 4.**
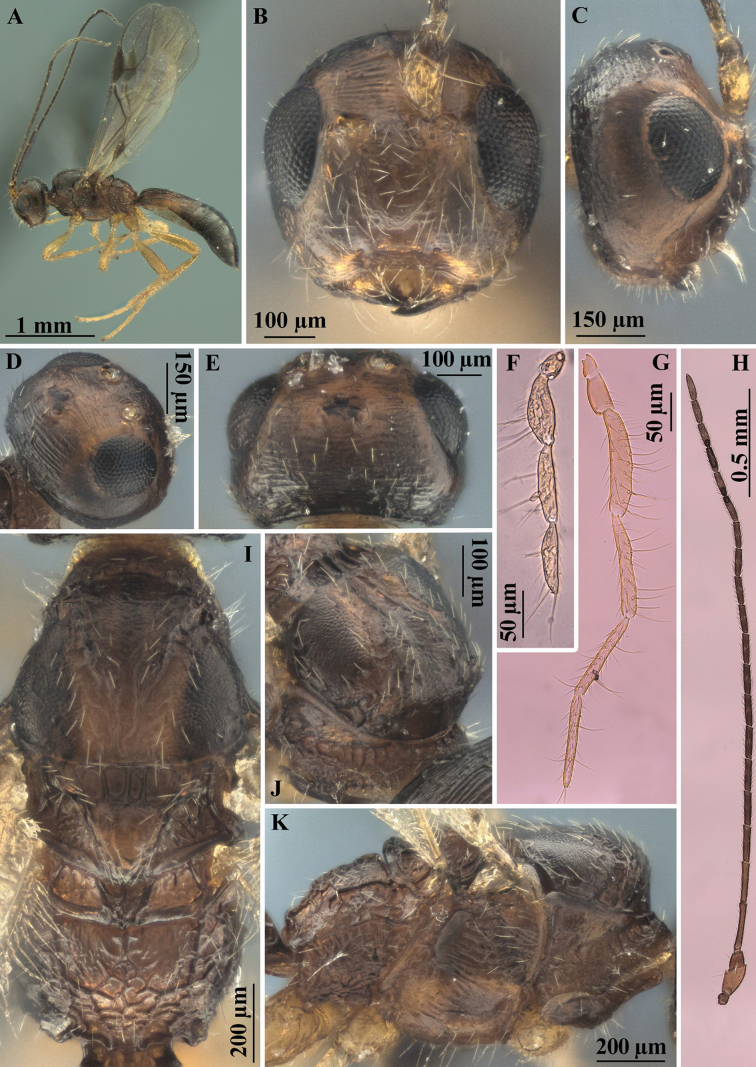
*Heterospilussicanus* (Marshall, 1888) (male) **A** habitus, lateral view **B** head, front view **C** head, lateral view **D** head dorso-lateral view **E** head dorsal view **F** labial palpus **G** maxillary palpus **H** antenna **I** mesosoma, dorsal view **J** pronotum, antero-dorsal view **K** mesosoma, lateral view.

**Figure 5.**
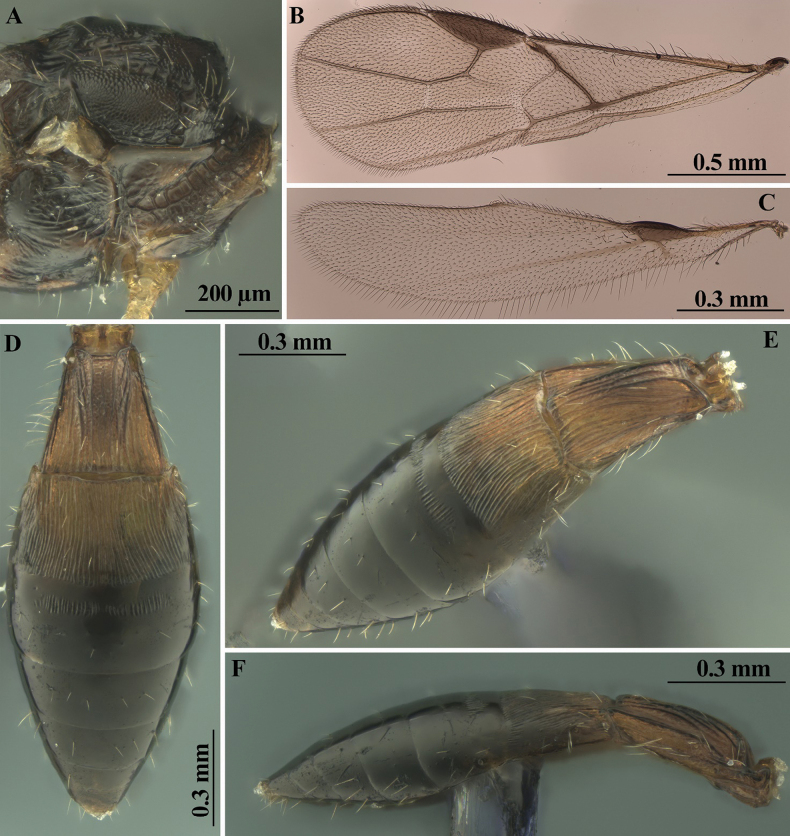
*Heterospilussicanus* (Marshall, 1888) (male) **A** pronotum and propleuron, dorso-lateral view **B** fore wing **C** hind wing **D** metasoma, dorsal view **E** metasoma dorso-lateral view **F** metasoma, lateral view.

Figs 1 , 2 , 3 , 4 , 5

Dendrosoter sicanus Marshall, 1888: 243.
Heterospilus sicanus : Tobias 1971: 194; 1976: 35; Shenefelt and Marsh 1976: 1312; Yu et al. 2016.
Atoreuteus ceballosi Docavo Alberti, 1960: 33, syn. nov.
Heterospilus ceballosi : Shenefelt and Marsh 1976: 1302; Yu et al. 2016.

###### Type material examined.

***Holotype* of *Dendrosotersicanus***: female, Italy, “Type” (round with red border), “*sicanus* Marsh. (Sicily)” (handwriting), “Marshall coll. 1904–120”, “Almost certainly type of *Dendrosotersicanus* Msh., G. Nixon, 25.I.38” (handwriting by G. Nixon), “This is definitely type of *Dendrosotersicanus* Marshall. Paul M. Marsh, VI–17–71” (handwriting by P. Marsh), “B.M. Type Hym. 3c.1751”, “NHMUK 010880780” (NHMUK, London). ***Holotype* of *Atoreuteusceballosi***: female, Spain, “Tenerife, Bajamar, 8.V.1901”, “♀”, “*Atoreuteusceballosi* Docavo n. sp.”, “Tipo”, “*Heterospilus ♀ ceballosi* Doc., det. Papp J., 1983”, “MNCN Cat. Typos N 11.246” (MNCN, Madrid).

###### Additional material examined.

Italy: Sicily, “Ottavio Ziino” Law History Library of the Law Department of the University of Palermo, 5.VI.2023 (E. Peri, S. Savoldelli, C. Jucker and S. Guarino), 18 females, 15 males (SAAF-UNIPA); Sicily, Vittoria, IX – X.1899 (G. Mantero), 1 female (ZISP). Russia: Crimea, Sebastopol, 5.V.1917 (W. Pliginski), 8 females with the same label of the latter (ZISP).

###### Redescription.

**Female (holotype).** Body length 2.6 mm; fore wing length 2.3 mm.

***Head*.** Head not depressed, its width 1.6× median length, 1.1× width of mesoscutum. Head behind eyes weakly convex anteriorly, evenly and roundly narrowed posteriorly. Transverse diameter of eye 1.2× longer than temple (dorsal view). Ocelli small, in almost equilateral triangle. POL 1.3× Od, 0.35× OOL. Diameter of antennal socket equal to distance between sockets, twice distance between socket and eye. Eye with sparse and short setae, without emargination opposite antennal sockets, 1.2× as high as broad. Malar space 0.7× height of eye, 1.2× basal width of mandible. Face convex, its width 1.2× height of eye and almost equal to height of face and clypeus combined. Malar suture absent. Clypeus with distinct lower flange. [Hypoclypeal depression covered by glue.] Occipital carina complete dorsally, ventrally joining hypostomal carina distant from base of mandible. Head below eyes (front view) roundly narrowed. Hypostomal flange distinct but narrow.

***Antennae*.** Antenna slender, filiform, 20-segmented, almost as long as body. Scape rather short and thick, 1.5× longer than its maximum width. First flagellar segment slender, almost straight, subcylindrical, 5.5× longer than apical width, almost as long as second segment. Penultimate segment 3.3× longer than wide, 0.6× as long as first segment, 0.9× as long as apical segment; the latter pointed apically and without spine.

***Mesosoma*.** Mesosoma not depressed dorso-ventrally, its length 1.6× maximum height. Pronotal neck rather long, dorsally without convex lobe, with rather distinct submedial pronotal carina; side of pronotum with distinct, almost straight, and rather wide submedian oblique crenulate furrow. Mesoscutum highly and perpendicularly elevated above pronotum, maximum width of mesoscutum 1.3× its median length. Median lobe of mesoscutum (dorsal view) protruding forwards, weakly convex anteriorly, with distinct and almost pointed anterolateral corners. Notauli wide, rather deep, densely and coarsely crenulate. Prescutellar depression deep, wide, with 4 carinae, finely sculptured between carinae, ~ 0.3× as long as wide, 0.45× as long as scutellum. Scutellum convex, with fine lateral carinae, its width 1.1× median length. Subalar depression rather deep, wide, sparsely and coarsely rugose-striate. Precoxal sulcus deep, almost straight, rugulose, running along anterior 0.6 of lower part of mesopleuron. Metanotal tooth (lateral view) relatively long, wide, distinctly pointed apically. Metapleural lobe rather large, more or less wide, rounded posteriorly. Propodeum (lateral view) regularly convex-roundly slanted from base to apex, without lateral tubercles; propodeal spiracle small.

***Wings*.** Fore wing 3.0× longer than its maximum width, 0.9× as long as body. Pterostigma 3.0× longer than wide. Radial vein (r) arising before middle of pterostigma, distance from base of pterostigma to radial vein (r) 0.85× distance from radial vein (r) to apex of pterostigma. Radial (marginal) cell not shortened. Metacarp (1-R1) 1.2× longer than pterostigma. First radial abscissa (r) almost as long as maximum width. Second radial abscissa (3-SR) as long as first abscissa (r), 0.25× as long as the straight third abscissa (SR1), 0.5× as long as trace of first radiomedial vein (2-SR). Trace of first radiomedial vein (2-SR) 2.3× longer than second radiomedial vein (r-m), 4.0× longer than recurrent vein (m-cu). Recurrent vein (m-cu) distinctly postfurcal. First medial abscissa (1-SR+M) curved. Discoidal (discal) cell 1.6× longer than its width. Distance (1-CU1) from nervulus (cu-a) to basal vein (1-M) ~ 0.5 of nervulus (cu-a) length; nervulus (cu-a) straight and almost perpendicular to longitudinal anal vein (1-1A). Mediocubital vein (M+CU1) almost straight. Parallel vein (CU1a) distinctly curved subbasally. Brachial (subdiscal) cell widely open distally, brachial vein (CU1b) absent. Hind wing 4.2× longer than wide. First abscissa of costal vein (C+SC+R) 1.2× longer than second abscissa (1-SC+R); second abscissa (1-SC+R) strongly sclerotised. Last costal abscissa (SC+R1) 0.8× as long as first (C+SC+R) and second (1-SC+R) abscissae combined. Radial vein (SR) strongly desclerotised. Medial (basal) cell narrow, almost parallel-sided in its apical half, its length ~ 11.0× maximum width, almost 0.3× length of wing. First abscissa of mediocubital vein (M+CU) 0.8× as long as second abscissa (1-M). Recurrent vein (m-cu) unsclerotised, almost interstitial, straight, very weakly oblique toward base of wing.

***Legs*.** Fore tibia with several rather slender spines arranged in longitudinal line. Hind coxa with basoventral tubercle, 1.3× longer than its maximum width. Hind femur rather wide, without dorsal protuberance, 3.5× longer than wide. Hind tarsus 0.9× as long as hind tibia. Basitarsus not thickened, without ventral keel, 0.5× as long as second–fifth segments combined. Second tarsal segment 0.7× as long as basitarsus, 1.6× longer than fifth segment (without pretarsus).

***Metasoma*.** Metasoma 0.9× as long as head and mesosoma combined, 1.8× longer than its maximum width. First segment with short acrosternite. First tergite with not high but rather distinct and wide median area, with distinct dorsope, without spiracular tubercles; tergite distinctly and almost linearly widened from base to apex. Length of first tergite equal to its apical width, 1.4× length of propodeum; maximum width of tergite ~ 2.0× its minimum width. Median length of second tergite 0.45× its basal width, 0.8× length of third tergite. Combined length of second and third tergites 0.9× basal width of second tergite, 0.7× their maximum width. Second suture present, but fine, usually weakly curved laterally. Third tergite without transverse furrow. Ovipositor sheath rather slender, 0.8× as long as metasoma, 1.1× longer than mesosoma, 0.4× as long as body, 0.5× as long as fore wing.

***Sculpture and pubescence*.** Vertex entirely distinctly and rather densely transversely striate, partly with very fine additional reticulation between striae; frons entirely densely and distinctly transversely striate [face covered in glue]; temple smooth. Mesoscutum densely and distinctly granulate, medioposteriorly with two posteriorly convergent carinae. Scutellum granulate. Mesopleuron entirely rugose-striate. Metapleuron entirely distinctly rugose-reticulate. Propodeum with rather wide, short and finely granulate-coriaceous basolateral areas, weakly delineated by carinae; areola finely delineated; basal carina 0.8× as long as anterior fork of areola; posterior 0.7 of propodeum irregularly rugose-reticulate. Hind coxa densely granulate, transversely striate dorsally. Hind femur finely and densely granulate-coriaceous. First tergite with rather distinct and posteriorly convergent dorsal carinae, densely and distinctly longitudinally striate, with fine and dense additional reticulation between striae. Second tergite mostly distinctly longitudinally striate, laterally smooth over rather wide area, rugulose postero-medially. Remaining tergites smooth. Vertex almost entirely with rather dense, short and semi-erect setae arranged in rows. Mesoscutum with rather dense, relatively long and semi-erect pale setae at wide area along notauli and scattered across lobes, all lobes narrowly glabrous medially. Hind tibia dorsally with short, sparse, semi-erect setae; length of these setae ~ 0.3× maximum width of hind tibia.

***Colour*.** Body reddish brown, vertex, mesonotum, and posterior half of metasoma dark reddish brown. Antenna dark brown, four basal segments pale brown. Palpi yellow. Legs entirely pale brown. Ovipositor sheath mainly brown, black apically. Fore wing very faintly infuscate. Pterostigma almost entirely brown.

***Variation*.** Head width 1.4–1.6× median length. Transverse diameter of eye 1.1–1.3× longer than temple (dorsal view). Malar space 0.6–0.7× height of eye. Hypoclypeal depression round, its width 0.7–0.8× distance from margin of depression to margin of eye, 0.4–0.5× width of face. Face mainly smooth. Antennae 20-segmented. First flagellar segment 4.5–5.5× longer than its apical width. Penultimate segment 3.0–3.3× longer than wide. Mesosoma length 1.6–1.7× maximum height. Maximum width of mesoscutum 1.2–1.3× its median length. Mesopleuron sometimes smooth in small submedial area. Basal carina of propodeum 0.8–1.0× as long as areola anterior fork. Wings. Pterostigma 2.8–3.6× longer than wide. Second radial abscissa (3-SR) 1.0–1.3× as long as first abscissa (r), 0.25–0.30× as long as third abscissa (SR1), 0.5–0.7× as long as trace of first radiomedial vein (2-SR). Discoidal (discal) cell 1.6–1.8× longer than its width. Legs. Hind femur 3.5–3.8× longer than wide. Hind tibia dorsal setae 0.3–0.4× maximum width of hind tibia. First metasomal tergite 1.3–1.4× longer than propodeum. Median length of second tergite 0.40–0.45× its basal width, 0.7–0.8× length of third tergite. Ovipositor sheaths 0.7–0.9× as long as metasoma, 1.1–1.2× longer than mesosoma. Body mainly dark reddish brown, sometimes ventrally distinctly paler. Legs entirely pale brown or yellow, anterior half of metasoma often yellow or pale reddish brown. Fore wing very faintly infuscate. Pterostigma brown or pale brown.

**Male.** Body length 2.6–2.8 mm; fore wing length 2.1 mm. Antennae slender, filiform, 21-segmented, approximately as long as body. Hind wing with relatively small, complex, brown stigma-like enlargement, its length 0.7–0.8× distance from base of wing to base of enlargement. Length of first metasomal tergite 1.1× its apical width. Second tergite entirely striate. Median length of second tergite 0.75× its anterior width, 1.2× length of third tergite. Third tergite with shallow and crenulate transverse furrow in anterior one–third. Body mainly brown to dark brown, anterior third of metasoma paler. Otherwise similar to female.

###### Host.

Until recently, the only reported host of this species was *Cryphaluspiceae* (Ratzeburg, 1837) (Coleoptera, Curculionidae, Scolytinae) (Tobias 1971; Kenis et al. 2004; Wegensteiner et al. 2015). However, the first author checked material assigned to this species deposited in the collection of the Zoological Institute RAS (St Petersburg, Russia) determined by Dr V. I. Tobias as *H.sicanus* (Marshall) (Tobias 1971). This sample comprised seven females and ten males with the label: “Teberda, Sev. Kavkaz [North Caucasus, Karachay-Cherkess Republic], on *Cryphaluspicaae*, T. Guryanova [leg], 24 VI [19]64”, “*Dendrosotinussicanus* Marsh., Tobias det. 1965”. Our redetermination of these specimens showed that they actually belong to another genus and species, Dendrosotinus (Gildoria) similis Boucek, 1955. Thus, the host of *H.sicanus* was unknown before this study and *Gastralluspubens* Fairmaire, 1875 (Coleoptera, Anobiidae) is the first and only known host of *H.sicanus*.

###### Distribution.

According to Taxapad, the world catalogue of Ichneumonoidea (Yu et al. 2016), besides Italy (Sicily), *H.sicanus* has also been recorded in Spain (Falco Gari et al. 1993), Croatia (Papp 1977), Serbia (Brajkovic 1989), and Hungary (Papp 1984); however, at least some of these records require confirmation. In Russia, this species has only been found in Crimea (new record; see ‘Additional material examined’), whereas its records from the North Caucasus of Russia (Tobias 1971, 1976) were erroneous (for details see ‘Remarks’ under the ‘Hosts’ section).

###### Comparative diagnosis.

*Heterospilussicanus* (Marshall) is very similar to *H.corsicus* (Marshall, 1888), but differs from the latter by having the head behind the eyes convex anteriorly and roundly narrowed posteriorly (evenly roundly narrowed posteriorly in *H.corsicus*), eyes setose, transverse diameter in dorsal view 1.1–1.3× length of temple (glabrous, transverse diameter 1.5–1.6× length of temple in *H.corsicus*), antenna slender (thickened in *H.corsicus*), mesosoma 1.6–1.7× longer than its height (1.8× in *H.corsicus*), medial lobe of mesoscutum without pointed anterolateral corners (with pointed corners in *H.corsicus*), radial vein (r) of fore wing arising slightly before middle of pterostigma (almost from or behind middle in *H.corsicus*), setae on dorsal side of hind tibia short, ~ 0.3× as long as maximum width of tibia (long, 0.5–0.7× in *H.corsicus*), and pterostigma almost entirely brown (yellow in *H.corsicus*).

Western Palaearctic *Heterospilus* species with an almost entirely sculptured vertex can be differentiated using the key below.

##### ﻿ Heterospilus corsicus

Taxon classificationAnimalia

(Marshall, 1888)

E41ABFB0-5DCA-53CE-A6AA-B128958C29D3

Fig. 6

Telebolus corsicus Marshall, 1888: 202.
Heterospilus corsicus : Shenefelt and Marsh 1976: 1303; Yu et al. 2016.
Caenophanes cingulatus Szépligeti, 1900: 213.
Heterospilus cingulatus : Shenefelt and Marsh 1976: 1302; Belokobylskij and Tobias 1986: 33 (as synonym); Yu et al. 2016.

###### Type material examined.

***Holotype* of *Teleboluscorsicus***: female, France (Corsica), “Type” (round with red border), “Corsica”, “B.M. Type Hym. 3c.188”, “*corsicus* Marsh.”, “Marshall coll. 1904–120.”, “B.M. Type Hym. *Teleboluscorsicus*Marshall 1888”. “NHMUK010880788” (NHMUK, London). ***Holotype* of *Caenophanescingulatus***: female, “Szóváta, Csiki”, “Transsylvania”, “Holotypus ♀ *Caenophanescingulatus* Szép., 1900 sp. n. / des/ Papp J. 1967”, “Hym. Typ. N 598. Museum Budapest”, “*Heterospiluscingulatus* Sz., det. Papp J., 1983” (HNHM).

**Figure 6.**
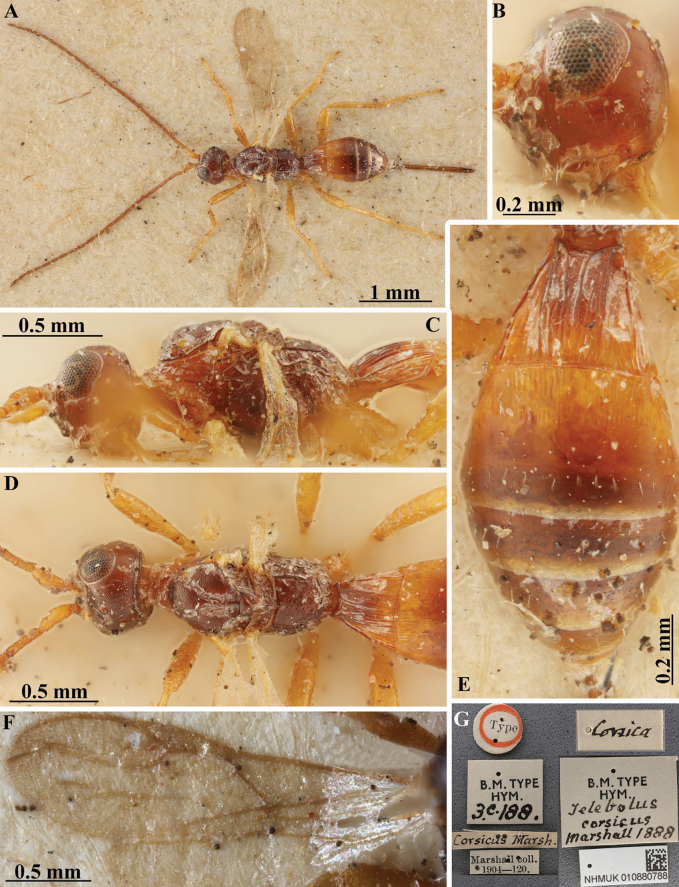
*Heterospiluscorsicus* (Marshall, 1888) (female, holotype) **A** habitus, dorsal view **B** head, lateral view **C** head, mesosoma and base of metasoma, lateral view **D** head, mesosoma and base of metasoma, dorsal view **E** metasoma, dorsal view **F** wings **G** labels.

###### Redescription.

***Female (holotype).*** Body length 2.6 mm; fore wing length 1.8 mm.

***Head*.** Head not depressed, its width 1.5× median length, 1.3× width of mesoscutum. Head behind eyes evenly and roundly narrowed. Transverse diameter of eye 1.6× longer than temple (dorsal view). Ocelli small, in almost equilateral triangle. POL almost equal to Od, 0.4× OOL. Diameter of antennal socket 1.3× distance between sockets, 1.8× distance between socket and eye. Eye without setae, with shallow emargination opposite antennal sockets, 1.2× as high as broad. Malar space 0.5× height of eye, 1.3× basal width of mandible. Face convex, its width 1.15× height of eye and 1.3× height of face and clypeus combined. Malar suture absent. Clypeus with short lower flange. Hypoclypeal depression rather small and suboval, its width 0.7× distance from edge of depression to eye, 0.35× width of face. Occipital carina complete dorsally, joining hypostomal carina ventrally distant from upper base of mandible. Head below eyes distinctly and weakly-roundly narrowed. Hypostomal flange distinct but narrow.

***Antenna*.** Antenna weakly thickened, filiform, 21-segmented, slightly longer than body. Scape rather long and thick, 1.5× longer than its maximum width. First flagellar segment weakly thickened, weakly curved, subcylindrical, 4.5× longer than its apical width, almost as long as second segment. Penultimate segment 2.7× longer than wide, 0.6× as long as first segment, 0.9× as long as apical segment; the latter pointed apically and without spine.

***Mesosoma*.** Mesosoma not depressed, its length 1.8× maximum height. Pronotal neck rather long, dorsally weakly convex, but without convex lobe and pronotal carina; side of pronotum with rather shallow, weakly curved and wide submedian oblique and sparsely crenulate furrow. Mesoscutum highly and roundly elevated above pronotum (lateral view), maximum width of mesoscutum 1.3× its length (dorsal view). Median lobe of mesoscutum (dorsal view) weakly protruding forwards, without anterolateral corners, distinctly convex anteriorly. Notauli rather narrow, deep, sparsely and finely crenulate. Prescutellar depression deep, long, with median carina, finely and irregularly sculptured, 0.5× as long as wide, 0.55× as long as scutellum. Scutellum convex, with fine lateral carinae, its basal width almost equal to median length. Subalar depression rather deep, wide, sparsely and coarsely striate. Precoxal sulcus rather deep, almost straight, distinctly crenulate, running along anterior 0.6 of lower part of mesopleuron. Metanotal tooth (lateral view) rather long, wide, more or less pointed apically. Metapleural lobe rather long, more or less wide, rounded apically. Propodeum (lateral view) regularly convex-roundly slanted from base to apex, without lateral tubercles; propodeal spiracle small.

***Wings*.** Fore wing 3.5× longer than its maximum width, 0.7× as long as body. Pterostigma 3.5× longer than wide. Radial vein (r) arising almost from middle of pterostigma, distance from base of pterostigma to radial vein (r) almost equal to distance from radial vein (r) to apex of pterostigma. Radial (marginal) cell not shortened. Metacarp (1-R1) 1.3× longer than pterostigma. First radial abscissa (r) 0.75× as long as maximum width of pterostigma. Second radial abscissa (3-SR) twice longer than first abscissa (r), 0.35× as long as the straight third abscissa (SR1), 0.7× as long as the trace of first radiomedial vein (2-SR). Trace of first radiomedial vein (2-SR) 2.2× longer than second radiomedial vein (r-m), 2.2× longer than recurrent vein (m-cu). Recurrent vein (m-cu) postfurcal. First medial abscissa (1-SR+M) almost straight. Discoidal (discal) cell 1.6× longer than wide. Nervulus (cu-a) almost interstitial, straight and subperpendicular. Mediocubital vein (M+CU1) almost straight. Parallel vein (CU1a) very weakly curved subbasally. Brachial (subdiscal) cell widely open distally, brachial vein (CU1b) absent. Hind wing with 5.5× longer than wide. First abscissa of costal vein (C+SC+R) 1.5× longer than second abscissa (1-SC+R); second abscissa (1-SC+R) strongly sclerotised. Last costal abscissa (SC+R) 0.75× as long as first (C+SC+R) and second (1-SC+R) abscissae combined. Radial vein (SR) very strongly desclerotised. Medial (basal) cell narrow, almost parallel-sided to weakly narrowed in apical half, its length 8.5× maximum width, ~ 0.3× length of wing. First abscissa of mediocubital vein (M+CU) almost as long as second abscissa (1-M). Recurrent vein (m-cu) unsclerotised, almost interstitial, straight, distinctly oblique toward base of wing.

***Legs*.** Fore tibia with several slender spines arranged in narrow stripe. Hind coxa with basoventral tubercle, ~ 1.4× longer than its maximum width. Hind femur relatively narrow, without dorsal protuberance, 4.0× longer than wide. Hind tarsus 0.9× as long as hind tibia. Basitarsus not thickened, without ventral keel, 0.5× as long as second–fifth segments combined. Second tarsal segment 0.7× as long as basitarsus, 1.3× longer than fifth segment (without pretarsus).

***Metasoma*.** Metasoma 0.9× as long as head and mesosoma combined, almost twice as long as its maximum width. First tergite with rather high and wide median area, with small dorsope, without spiracular tubercles; tergite strongly and almost linearly widened from anterior to posterior apex. Length of first tergite 0.85× its apical width, a little larger than length of propodeum; maximum width of tergite 2.5× its minimum width. Median length of second tergite 0.4× basal width of second tergite, 0.7× length of third tergite. Combined length of second and third tergites 0.9× basal width of second tergite, 0.7× their maximum width. Second suture distinct, distinctly curved laterally. Third tergite without sculptured transverse furrow. Ovipositor sheaths 0.8× as long as metasoma, 1.1× longer than mesosoma, 0.4× as long as body, 0.6× as long as fore wing.

***Sculpture and pubescence*.** Vertex rather finely and densely transversely striate with additional rugulosity between striae; frons mostly finely and densely transversely striate. Face mainly smooth, rugose medially; temple finely striate above, but mostly smooth. Mesoscutum densely and rather finely granulate, with two carinae medioposteriorly. Scutellum finely granulate. Mesopleuron entirely finely reticulate-granulate. Metapleuron entirely and rather distinctly rugose-reticulate. Propodeum with rather wide and finely granulate-coriaceous basolateral areas, distinctly delineated by carinae; areola indistinctly delineated by carinae; basal carina relatively long, 0.8× as long as anterior fork of areola; posterior 0.7 of propodeum coarsely and irregularly rugose-reticulate. Hind coxa densely granulate, coarsely transversely striate dorsally. Hind femur finely coriaceous. First tergite with rather distinct and convergent posteriorly dorsal carinae, distinctly longitudinally striate, with very fine, partly indistinct ground sculpture. Second tergite entirely densely and distinctly longitudinally striate. Remaining tergites smooth. Vertex partly with rather sparse and relatively long setae, almost glabrous medially. Mesoscutum with sparse, rather long and semi-erect pale setae arranged almost in one line along notauli and marginally, all lobes widely glabrous medially. Metapleuron widely glabrous medially. Hind tibia dorsally with relatively long, sparse and semi-erect setae; length of these setae 0.5–0.7× maximum width of hind tibia.

***Colour*.** Body dark reddish brown, almost black; pronotum pale reddish brown; first (except its dark medio-anterior half), second and anterior half of third metasomal tergites pale brown with reddish tint. Antenna dark reddish brown, paler basally. Palpi yellow. Legs pale brown. Ovipositor sheath almost black. Fore wing faintly infuscate. Pterostigma yellow.

###### Distribution.

France (Corsica), Italy, Hungary, Romania, Bulgaria, Moldova, Crimea (Yu et al. 2016).

###### Remarks.

The records of this species for Lithuania, Kazakhstan, Korea, and the Russian Far East by Belokobylskij and Tobias (1986) were erroneous due to the unclear understanding of this species before the study of the holotype.

###### Host.

Unknown.

### ﻿Key to the Western Palaearctic species of *Heterospilus* with distinctly sculptured vertex

**Table:** 

1	Metasoma behind third tergite striate anteriorly on fourth or fourth and fifth tergites	**2**
–	Metasoma entirely smooth behind third tergite; often third tergite also smooth	**6**
2	Fore wing strongly shortened, reaching at maximum to middle of metasoma; wing venation in distal half of fore wing strongly reduced. [Europe (rarely), Turkey, Iran, Mongolia]	***H.hemipterus* (Thomson, 1892) (*Lituaniabrachyptera* Jakimavicius, 1968)**
–	Fore wing not shortened, complete, prolonged behind posterior end of metasoma; wing venation in distal half of fore wing complete as usual for *Heterospilus*	**3**
3	First metasomal tergite relatively long, its length not less than posterior width. Often only fourth tergite striate anteriorly. Vertex often less distinctly striate. Body slender and slim	**4**
–	First metasomal tergite short, its length distinctly shorter than posterior width. Fourth and fifth tergites always striate anteriorly. Vertex distinctly and coarsely transversely striate. Body robust	**5**
4	Second tergite shorter, its medial length ~ 0.3× anterior width. Sculpture of first two tergites distinctly striate, less infilled with rugosity. Ovipositor projecting just more than 0.6× length of metasoma, just more than 1.1× length of hind tibia. Body predominantly reddish brown. [Europe, Russia (widely), western and central Asia, China, Korea, Japan]	***H.leptosoma* Fischer, 1960**
–	Second tergite longer, its medial length ~ 0.4× anterior width. Sculpture of first two tergites less regular, more rugulose. Ovipositor shorter, projecting 0.3–0.5× length of metasoma, 0.7–1.1× length of hind tibia. Body usually extensively dark. [UK, Sweden, China (?)]	***H.fuscexilis* M. Shaw, 1997**
5	Ovipositor sheaths distinctly shorter than metasoma, 0.4–0.6× as long as metasoma. Body often predominantly brownish yellow or light reddish brown. [Holarctic]	***H.cephi* Rohwer, 1925 (*H.testaceus* Telenga, 1941; *H.rubicundus* Fischer, 1960; Rhaconotusollivieri(Giraud)var.flavaFahringer 1931, syn. nov.)**
–	Ovipositor sheaths slightly shorter than or almost equal to metasoma, 0.7–1.0× as long as metasoma. Body often predominantly reddish brown to light reddish brown with often dark propodeum and first metasomal tergite. [Europe, Russia (widely), Turkey, Israel, Iran, Kazakhstan, Mongolia, China, Korea, Japan]	***H.tauricus* Telenga, 1941 (*H.graeffei* Fischer, 1960)**
6	Second metasomal tergite striate only on its anterior one–third or quarter. Suture between second and third tergites absent. Mesopleuron smooth over wide median area. [Portugal (Madeira), Israel, Russia (Crimea)]	***H.divisus* (Wollaston, 1858)**
–	Second metasomal tergite striate or striate-rugulose over anterior fourth–fifths or entirely. Suture between second and third tergites present but sometimes rather weak. Mesopleuron usually rugose-reticulate over wide median area	**7**
7	Head behind eyes (dorsal view) weakly convex in anterior half and roundly narrowed in posterior half; transverse diameter of eye 1.1–1.3× longer than temple. Mesosoma 1.5–1.6× longer than maximum height. Medial lobe of mesoscutum without or with indistinct anterolateral corners. Radial vein (r) of fore wing arising before middle of pterostigma. [Spain, Italy, Croatia, Hungary, Russia (Crimea)]	***H.sicanus* (Marshall, 1888) (*Atoreuteusceballosi* Docavo Alberti, 1960, syn. nov.)**
–	Head behind eyes (dorsal view) evenly and rather distinctly roundly narrowed; transverse diameter of eye 1.5–2.0× longer than temple. Mesosoma 1.8–2.0× longer than maximum height. Medial lobe of mesoscutum usually with distinct pointed anterolateral corners. Radial vein (r) of fore wing arising from or slightly behind middle of pterostigma	**8**
8	Malar space 0.5–0.6× height of eye, 1.2–1.3× basal width of mandible. Occipital carina ventrally joining hypostomal carina. Precoxal sulcus distinctly crenulate. Second segment of hind tarsus 1.3–1.5× longer than fifth segment (without pretarsus). Third metasomal tergite without transverse furrow. Mesopleuron entirely reticulate-granulate. Basal carina of propodeum relatively long. Pterostigma yellow or light brown. [France (Corsica), Italy, Hungary, Romania, Bulgaria, Moldova, Ukraine]	***H.corsicus* (Marshall, 1888) (*Caenophanescingulatus* Szépligeti, 1900)**
–	Malar space 0.4× height of eye, equal to basal width of mandible. Occipital carina obliterated below and not joining hypostomal carina ventrally. Precoxal sulcus smooth. Second segment of hind tarsus almost 2.0× longer than fifth segment (without pretatsus). Third metasomal tergite with crenulate transverse furrow in anterior one–third. Mesopleuron smooth over lower three–fifths. Basal carina of propodeum very short. Pterostigma brown. [Spain]	***H.marchi* (Docavo Albert, 1960)**

## ﻿Discussion

The species *Dendrosotersicanus* Marshall, 1888 and *Teleboluscorsicus* Marshall, 1888 both actually belonging to the genus *Heterospilus*, were described in the same year and in the same book (Marshall 1888) and have never subsequently been redescribed or compared with each other. The diagnostic characters of these species were relatively badly designated and the reliable determination of these taxa as well as stable differences between the species subsequently caused certain difficulties. This study with illustrated redescriptions of the type material, together with the preparation of a key for determination of the European *Heterospilus* species with a sculptured vertex, should help to avoid errors in their identification.

The holotype (female) of *Atoreuteusceballosi* Docavo Albert, 1960, studied by the first co-author in MNCN (Madrid, Spain), is morphologically very similar to *H.sicanus* (Marshall, 1888), which allowed us to synonymise the first name under the second as a new synonym. Also in the same Museum (MNCN), the holotype of *Atoreuteus* (= *Heterospilus*) *marchi* Docavo Albert, 1960, described from Spain (female, with labels “Barcelona, 27.V.1896”, “♀”, “*Atoreuteusmarchi* Docavo n. sp.”, “Holotip”, “Heterospilus?tauricus Tel., det. Papp J., 1983”, “MNCN Cat. Typos N 11.489”), was examined, which helped us to evaluate the status of this species as closely related to *H.corsicus* (Marshall, 1888). The first co-author has also studied) the single specimen (female) in NHMW (Wien, Austria) of the form *Hormiopterus* (= *Rhaconotus*) *ollivieri* Giraud var.flavaFahringer 1931 (Fahringer 1931; Shenefelt and Marsh 1976) (with labels: “Hormiopterusolivieri (sic!) Gr var flava m.” (handwriting by Fahringer), “olivieri (sic!) Fer. (sic!), det. Fahringer”, but without any geographic information), which turned out to be a new junior synonym of *Heterospiluscephi* Rohwer, 1925.

The discovery of *H.sicanus* in the frass, holes, and tunnels of rare books damaged by *Gastralluspubens* Fairmaire (Coleoptera: Ptinidae), with its illustrated redescription and updated diagnosis, and its comparison with the morphologically similar *H.corsicus* sheds light on these rare and barely studied doryctine species. The inclusion of digital photographs and the key for determination of the Western Palaearctic species of *Heterospilus* with distinctly sculptured vertex have also helped to improve the precise species identification of these taxa. This accurate identification of parasitoids is crucial for effective and sustainable pest management programmes. In fact, *Heterospilussicanus* could be a potential biological control agent against *G.pubens*, which is an emerging threat to librarians and archivists in Italy and across Europe given the destructive larval activity of the beetles, which causes serious damages to books, especially ancient.

## Supplementary Material

XML Treatment for Heterospilus sicanus

XML Treatment for Heterospilus corsicus

## ﻿Additional information

### Author ORCIDs

Sergey A. Belokobylskij https://orcid.org/0000-0002-3646-3459

Salvatore Guarino https://orcid.org/0000-0001-6670-3997

Sara Savoldelli https://orcid.org/0000-0003-2689-4252

Costanza Jucker https://orcid.org/0000-0001-5052-3705

Ezio Peri https://orcid.org/0000-0002-1095-7258

Gavin R. Broad https://orcid.org/0000-0001-7223-5333

Giuliano Cerasa https://orcid.org/0000-0003-1837-4302

### Data availability

All of the data that support the findings of this study are available in the main text.
